# The Expression Pattern and Regulatory Mechanism of the G0/G1 Switch Gene 2 (*G0S2*) in the Pathogenesis and Treatment of AChR Myasthenia Gravis (MG)

**DOI:** 10.1155/2020/4286047

**Published:** 2020-09-30

**Authors:** Liqun Xu, Zhibin Li, Yi Li, Zhaohui Luo, Yuebei Luo, Bo Xiao, Huan Yang

**Affiliations:** Department of Neurology, Xiangya Hospital, Central South University, Changsha, Hunan 410008, China

## Abstract

This study is aimed at exploring the expression pattern and methylation level of *G0S2* in the peripheral blood mononuclear cells (PBMCs) of myasthenia gravis (MG) patients with positive acetylcholine receptor (AChR) autoantibodies and revealing the relationship between the *G0S2* methylation pattern and MG. The relationship between the NFAT family members and *G0S2* was explored to reveal the regulatory mechanism of *G0S2* in the pathogenesis and treatment of AChR MG. Moreover, we attempted to demonstrate the potential therapeutic mechanism of tacrolimus in AChR MG. The relative *G0S2* expression level in the PBMCs of healthy people was compared with that in the PBMCs of AChR MG patients with quantitative real-time PCR (qRT-PCR). The methylation frequency of the *G0S2* promoter was detected by bisulfite sequencing PCR (BSP) and pyrosequencing. A dual-luciferase reporter system was used to reveal the relationship between the *G0S2* promoter and nuclear factor of activated T cells 5 (*NFAT5*). The qRT-PCR results showed that *G0S2* expression was significantly upregulated in the B cells and CD8+ T cells of AChR MG patients but not in the CD4+ T cells, and these expression differences were significantly associated with a decrease in *G0S2* methylation. *NFAT5*, which was speculated to bind to island 1 (p1) in the *G0S2* promoter, may regulate the lymphocyte balance by regulating *G0S2* gene expression but failed to affect the methylation of the *G0S2* promoter. Tacrolimus therapy-induced methylation and overexpression of *NFAT5* could significantly reduce the expression of *G0S2* in AChR MG patients. The *G0S2* gene was remarkably upregulated in the PBMCs of MG patients. *NFAT5* may affect transcription initiation and downregulate *G0S2* expression through p1 in the promoter, thus controlling *G0S2* gene expression and regulating the lymphocyte balance. Therefore, *G0S2* could be an immune regulatory factor in both AChR MG occurrence and treatment with tacrolimus.

## 1. Introduction

Myasthenia gravis (MG) is an autoimmune disease characterized by a transmission disorder of the neuromuscular junction. The molecular immunopathology of MG is due to the presence of circulating autoantibodies that specifically target the acetylcholine receptor (AChR), muscle-specific tyrosine kinase (MuSK), or low-density lipoprotein (LDL) receptor-related protein 4 (LRP4). Patient-derived AChR, MuSK, and LRP4 autoantibodies in MG are demonstrably pathogenic (B cells in the pathophysiology of myasthenia gravis), and patients most often harbor only one of these autoantibody specificities. In approximately 75–85% of MG patients, AChR autoantibodies are detectable [1, 2]. The attack on postsynaptic components or functionally related molecules by AChR antibodies is regarded as the main pathogenesis of MG. Our understanding of MG immunopathology remains incomplete. It appears that the mechanism used by B cells for autoantibody production in AChR and MuSK MG differs, although details on both are needed to understand the immunopathology that will guide the development of more effective therapies [3].

It is generally agreed that the cell cycle plays an important role in regulating the clonal deletion of self-antigen-reactive lymphocytes, maintaining immune homeostasis and preventing the occurrence of autoimmune diseases [4]. Peripheral blood mononuclear cells (PBMCs) are suspended in blood plasma, which circulates these immune-related cells throughout the body [5]. Investigating PBMC disorders could represent a method of better understanding the pathogenesis of MG. Most MG patients could achieve satisfactory improvement via the application of immunosuppressive therapy, such as glucocorticoid [3]. However, the long-term use of corticosteroids is associated with severe adverse events. To prevent these side effects, a novel immunosuppressant is desired. Tacrolimus (FK-506), which is similar to cyclosporin A (CsA), is a kind of calcineurin inhibitor that can restrain calcium-dependent phosphatase calcineurin and suppress the immune response [6]. Compared to CsA, tacrolimus is more effective but has fewer side effects [7], and its benefits for MG treatment have been proven in several studies and recommended by international experts' consensus. However, until now, the therapeutic mechanism of tacrolimus on MG has not been elucidated.

A previous study suggested that cyclosporin A could downregulate the expression level of G0/G1 Switch Gene 2 (*G0S2*) in cultured human blood mononuclear cells [8]. Tacrolimus may share the same therapeutic mechanism with cyclosporin A in the treatment of MG. Thus, the *G0S2* may be a potential therapeutic site for tacrolimus. In a previous report, the *G0S2* gene was found in PBMCs and recognized as a potential tumor suppressor gene [9]. Studies on the cell cycle suggest that *G0S2* expression is related to the G0 to G1 transition; however, elevated levels of *G0S2* inhibited cell proliferation and the G0 to G1 transition [10]. Additionally, other studies have suggested that *G0S2* is significantly upregulated in the lymphocytes of immunodeficient and systemic lupus erythematosus patients [11, 12]. We screened the integration of aberrant lncRNA and mRNA expression changes in the PBMCs from MG patients in our previous study [13]. The lncRNA oebiotech_11933, which had 3 cis genes (*C1orf74*, *G0S2*, and *TRAF3IP3*), was one of the 12 differently expressed lncRNAs from the MG patients. The expression of the *G0S2* gene was significantly higher/lower in MG patients than in healthy volunteers at the mRNA level. Based on these findings, we focused on this gene and investigated the function of *G0S2*. Moreover, the promoter region of *G0S2* contains potential binding sites for nuclear factor of activated T cells (*NFAT*) [14]. Moreover, the binding sites were located in the promoter region at CpG sites, so the NFAT gene family members may affect the *G0S2* expression level by affecting methylation. In our study, we implemented a luciferase assay and confirmed the predicted that the inhibitory regulation of *NFAT5* and *G0S2* could be implicated in the pathophysiology of AChR MG. *NFAT5* encodes a transcription factor belonging to a family of proteins that plays a central role in regulating gene transcription during the immune response induced by osmotic stress in mammalian cells. *NFAT5* is vital to cell cycle progression and T cell proliferation [15].

In the present study, we aimed to explore the expression pattern and methylation level of *G0S2* in the PBMCs of AChR MG patients and reveal the relevant relationship between *G0S2* patterns and AChR MG. The relationships between *NFAT5* and *G0S2* in B cells and T cells were explored to reveal the different regulatory mechanisms in different lymphocyte subsets during the pathogenesis and treatment of MG. In addition, our future studies evaluating the effects of tacrolimus on CD8+ T cells and B cells in AChR MG patients should be particularly informative.

## 2. Material and Methods

### 2.1. Patients

This study was approved by the Ethics Committee of Xiangya Hospital (No. 201503233); all patients provided written informed consent. All experiments were performed according to the relevant guidelines and regulations. PBMC samples were obtained from MG patients with positive AChR antibodies and control patients without myopathy. Peripheral blood samples from 50 AChR MG patients and 30 healthy individuals were collected for gene expression analysis. All cell and tissue samples were used for the cell apoptosis assay and quantitative real-time PCR (qRT-PCR). MG patients ranged from 18 to 70 years old. All patients were initially treated as outpatients and did not take any immunosuppressants in the 3 months prior to the start of the study. They did not have any autoimmune diseases (ADs). The peripheral blood samples from the control group were collected from the medical examination center (MEC).

### 2.2. Reagents

TRIzol Reagent (Cat. No. 15596-018) and an anti-AmIg-FITC antibody were obtained from Invitrogen, Inc. SYBR® Premix Ex Taq™ (Perfect Real Time), and the Genomic DNA Extraction Kit were purchased from TaKaRa, Inc. (Otsu, Shiga, Japan). The ReverTra Ace® qPCR RT Kit was purchased from TOYOBO, Inc. The EpiTect Bisulfite Kit was purchased from Qiagen, Inc.

### 2.3. Monocyte Isolation

Approximately 5 mL of diluted peripheral blood (heparinized) was further diluted with Hank's buffer at a ratio of 1 : 3 and was slowly added to 5 mL lymphocyte separation medium (Percoll, Solarbio, Beijing) in a 15 mL centrifuge tube. The mononuclear leukocyte layer was isolated by centrifugation at 1200 rpm for 20 min. Cells at the interface were collected and slowly washed with 5 mL of sterile Hank's buffer and centrifuged at 1000 rpm for 5 min. Then, the cell pellets were washed and centrifuged 2-3 times with Hank's buffer using the same parameters. After that, magnetic beads were used to separate the CD4+ T cells from the CD19+ B cells. Total PBMCs from MG patients, PBMCs from healthy controls, CD4+ T cells from MG patients, and CD19+ B cells from MG patients were resuspended in DMEM complete medium (HyClone, USA) with 15% fetal bovine serum (Gibco, USA). The cell suspension was seeded in a culture flask (Corning, USA).

### 2.4. RNA Isolation and Quantitative Real-Time PCR

TRIzol Reagent (Invitrogen, Cat. No. 15596-018) was used to extract the total RNA from the peripheral blood and thymus tissues of MG subjects (with and without thymoma) and control patients according to the manufacturer's instructions. The total RNA concentrations were measured with a UV spectrophotometer. Reverse transcription was then performed with a ReverTra Ace® qPCR RT Kit (TOYOBO) after the genomic DNA was removed. (SYBR Premix Ex Taq™, TaKaRa, Otsu, Shiga, Japan) in triplicate with sequence-specific PCR primers on a StepOne Plus system (Applied Biosystems, Foster City, CA, USA). The *GAPDH* gene was used as an internal control for each sample. The primer sequences are listed in Table 1. PCR amplification for all detected genes was performed in triplicate under the following conditions: initial denaturation at 95°C for 30 s, followed by 40 cycles of 95°C for 5 s and 60°C for 34 s. The relative quantification of gene expression was normalized against *GAPDH* by using the 2^−ΔΔCT^ method.

### 2.5. Analysis of the *G0S2* Promoter Methylation

Genomic DNA was isolated from PBMCs. The present study selected the region from -666 bp to +31 bp of the *G0S2* genomic gene sequence as the target fragment. Bioinformatics analysis showed that there are two different CpG islands in the *G0S2* gene promoter region (island 1 (p1) and island 2 (p2) of the *G0S2* promoter). The primers for both p1 and p2 located in the *G0S2* promoter region were designed by the Methyl Primer Express version 1.0 software (Thermo Fisher Scientific, Inc.). The primer sequences are listed in Table 1.

Genomic DNA samples from the PBMCs of CD4+ T cells, CD8+ T cells, and CD19+ B cells from MG patients and healthy control, respectively, were extracted according to the instructions provided with the Genomic DNA Extraction Kit; then, the samples were analyzed by bisulfite sequencing PCR (BSP) with the EpiTect Bisulfite Kit, which detected the methylation status of the CpG islands at the *G0S2* gene promoter regions of all samples [16, 17]. PCR was used to amplify p1 and p2 in the *G0S2* promoter and was performed in a total volume of 50 *μ*L with the following reagents: 1 *μ*L cDNA template, 1 *μ*L G0S2-bsp-1F/R for p1 (or G0S2-bsp-2F/R for p2), 1 *μ*L DNA polymerase, 5 *μ*L 10× PCR buffer (Mg^2+^ plus), 4 *μ*L dNTP mixture, and 37 *μ*L RNase-free ddH_2_O. When the CpG sites in the region analyzed by methylation-specific PCR (MSP) are methylated, the methylated (M) band will appear, while the demethylated (U) band will be present when the sites are demethylated. Occasionally, both bands could be present if the sites are partially methylated. The PCR product was purified and cloned into a pMD-18T vector. The positive clones were selected and sequenced.

After confirming the exact sequence with traditional sequencing, the methylation status of the *G0S2* gene promoter region was further validated by pyrosequencing with an EpiTect Bisulfite Kit [18]. Genomic DNA from 64 MG patients and 64 healthy volunteers was modified with bisulfite reagents following the manufacturer's instructions (Zymo Research, Orange, CA). This modification resulted in a conversion of demethylated cytosine to thymine, whereas the methylated cytosine remained unchanged. A total of 20 ng of bisulfite-modified genomic DNA from each sample was subjected to PCR amplification and was directly pyrosequenced with the ABI 3700 automated sequencing system (Applied Biosystems, Carlsbad, CA) to detect the methylation level of each CpG site in the *G0S2* promoter. *G0S2* methylation was also detected by real-time quantitative-MSP (RQ-MSP) using SYBR Premix ExTaq™ according to the manufacturer's instructions to verify the pyrosequencing result. The normalized ratio (NM-G0S2) was used to assess the *G0S2* methylation in each sample and was determined using the following formula:

(1) $$\begin{array}{c} N_{\text{M}‐\text{G}0\text{S}2}={\left(E_{\text{M}‐\text{G}0\text{S}2}\right)}^{\Delta \text{CT}\cdot \text{G}0\text{S}2\left(\text{control}‐\text{sample}\right)}÷{\left(E_{\text{ALU}}\right)}^{\Delta \text{CT}\cdot \text{ALU}\left(\text{control}‐\text{sample}\right)}. \end{array}$$

### 2.6. Regulatory Relationship Detection between *NFAT5* and the *G0S2* Gene

To verify how the *NFAT5* gene regulates the expression level of *G0S2*, the *NFAT5* gene was cloned and sequenced with sequence-specific PCR primers, and the recombinant plasmid pEGFP-N1-NFAT5 was constructed. Different concentrations of pEGFP-N1-NFAT5 (100 ng and 200 ng) were transfected into the PBMCs of MG patients with lipofectamine (Lipo 2000). The control group was transfected with scrambled *G0S2* mRNA constructs, and *G0S2* promoter methylation was analyzed 48 hours after the transfection. The methylation statuses were further validated by BSP. The primer sequences used are listed in Table 1. *GAPDH* was used as an internal control. The relative quantification of gene expression was normalized against *GAPDH* by using the 2^−ΔΔCT^ method.

### 2.7. Detecting the Relationship between *NFAT5* and the *G0S2* Gene Promoter

Dual-luciferase reporters are often employed to make relational or radiometric measurements within one experimental system. Typically, one reporter is used as an internal control, and the other reporter is normalized. To identify how the *NFAT5* gene and the promoter of *G0S2* interact, the recombinant plasmids pGL3-Basic-p1 (inserted with p1), pGL3-Basic-p2 (inserted with p2), pGL3-Basic-p12 (inserted with the continuous p1 and p2 sequences), and pEGFP-N1-*NFAT5* (inserted with *NFAT5*) were constructed.

Jurkat T cells were incubated in DMEM containing 10% fetal bovine serum and placed in an incubator at 37°C and 5% CO_2_. Cells in the logarithmic growth phase were counted with trypan blue and seeded in 24-well cell culture plates at 1∗10^5^/well until the cells grew to 80% confluence. Then, 750 ng of the reporter plasmids pGL3-Basic-p1, pGL3-Basic-p2, and pGL3-Basic-p12 and 60 ng of pRL-TK were diluted with 50 *μ*L serum-free OPT1-DMEM and incubated for 5 min at room temperature. Liposomes were also cultured in 50 *μ*L serum-free OPT1-MEM with 2 *μ*L of Lipofectamine™ 2000. This plasmid and liposome mixture were incubated at room temperature for 5 min, gently mixed, and allowed to stand for 20 min at room temperature. The original medium in a 24-well plate was discarded; the plate was washed twice with PBS, and 500 *μ*L serum-free OPT1-DMEM was added per well. Cells were transfected with the plasmid and liposome mixture and were gently shaken and mixed. The transfection mixture was placed in a 37°C, 5% CO_2_ incubator. After transfection for 24 hours, the transfected cells were subjected to a luciferase assay to detect the hLuc and hRluc activities [19, 20]. When cells positive for pGL3-Basic-p1, pGL3-Basic-p2, and pGL3-Basic-p12 were identified, liposomes, Lipofectamine™ 2000 (2 *μ*L), and 200 ng pEGFP-N1-*NFAT5* were mixed with 50 *μ*L serum-free OPT1-MEM at room temperature for 20 min. The plasmid (pEGFP-N1-*NFAT5*) and liposome mixture was transfected into 3 positive cell lines and was gently shaken and mixed. The mixture was placed in a 37°C, 5% CO_2_ incubator. After transfection for 24 hours, the transfected cells were subjected to a luciferase assay to detect the hLuc and hRluc activities [19, 20]. Each luciferase assay was performed in triplicate, and GraphPad Prism 4.0 was used to analyze the data and generate the histograms.

### 2.8. Regulation of the *G0S2* Expression Level

During a 3-month course of tacrolimus therapy in MG patients, the subjects received daily doses of 3-5 mg tacrolimus prior to eating [8]. The dose of tacrolimus to treat MG ranges from a fixed daily dose of 3 mg to a weight-based approach of 0.05 to 0.1 mg/kg/day. Peripheral blood samples were collected from MG patients before and after tacrolimus therapy for RNA extraction and qRT-PCR. The expression levels of *G0S2* in the PBMCs with and without tacrolimus therapy for three months were measured by qRT-PCR.

Total PBMCs were isolated from MG patients and resuspended in DMEM complete medium+15% fetal bovine serum and 1% penicillin/streptomycin in a culture flask (Corning, USA). Isolated PBMCs were treated as follows: PBMCs were cocultured with 5-aza-dC (DAC) to perform DNA demethylation; the original PBMCs were transfected with the plasmid (pEGFP-N1-*NFAT5*) and liposome mixture; and PBMCs were treated with DAC and transfected with the plasmid (pEGFP-N1-*NFAT5*) and liposome mixture. All different cells were collected for RNA extraction and qRT-PCR. The expression levels of *G0S2* in PBMCs treated with different conditions were measured by qRT-PCR.

### 2.9. Statistical Analyses

All result data were statistically analyzed with SPSS 13.0 software. All data with continuous variables are expressed as the mean ± SD. Comparisons between two groups were performed by Student's *t* test. *p* < 0.05 was considered statistically significant.

## 3. Results

### 3.1. *G0S2* Gene Is Upregulated in MG Patients

**Figure 1:**
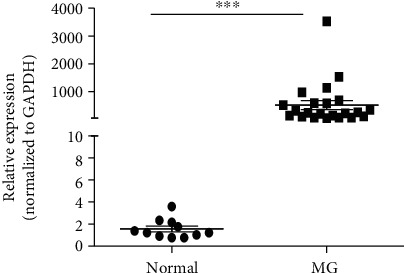
The relative expression level of *G0S2* gene in the PMBC from MG patients and normal healthy volunteers. ^∗∗∗^*p* < 0.01 vs. control. The *y*-coordinate represents the relative expression level, each dot represents an individual expression result, MG means the myasthenia gravis patients, and normal means healthy volunteers. The relative expression of *G0S2* was normalized to the expression level of internal control gene GAPDH.

Previous mRNA microarray analyses of the PBMCs of MG patients [13] revealed that distinct biological pathways had diverse functions. Based on the GO biological process results, differentially expressed genes between healthy people and MG patients were annotated. As a key gene in cell cycle regulation, the expression of the *G0S2* gene was significantly higher in MG patients than in healthy volunteers at the mRNA level. Based on these findings, we focused on this gene and investigated the function of *G0S2*. The expression patterns of *G0S2* in the PBMCs of 50 MG patients and 20 healthy volunteers were determined by qRT-PCR. Compared with the mRNA microarray analysis results, the *G0S2* gene showed a more significant upregulation in the peripheral blood of MG patients. qRT-PCR revealed that the expression level of *G0S2* was sharply upregulated (up to a 2,200-fold change) in the PBMCs of MG patients (Figure 1). The expression pattern of *G0S2* in the PBMCs of MG patients suggested the involvement of *G0S2* in MG.

### 3.2. Association between *G0S2* Methylation and Expression in MG Patients

**Figure 2:**
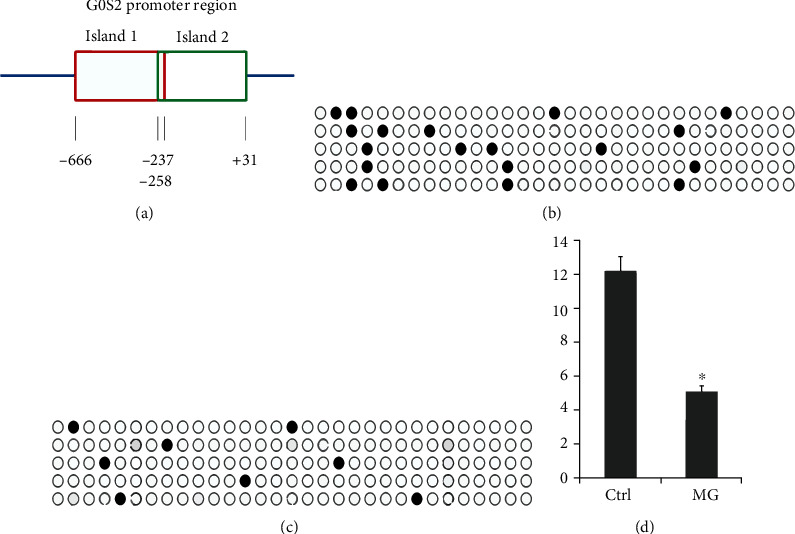
G0S2 methylation level in control and MG patients. (a) The positions of two different CpG islands in G0S2 promoter region. Box represents the different CpG islands. Number represents the positions of nucleobase. (b) G0S2 methylation density in the control group; blank circle means result which was not methylation; the black circle means methylation result. (c) G0S2 methylation density in the MG patients. Blank circle means result which was not methylated; the black circle means methylation result. (d) Normalized ratio of G0S2 methylation. MG means the myasthenia gravis patients; ctrl means healthy volunteers. The *y*-coordinate represents the rate of methylation result.

Through sequencing and bioinformatics analysis, two CpG islands (-258 bp to +31 bp and -666 bp to -237 bp) were found in the promoter region of *G0S2* (Figure 2(a)) and were named p1 and p2. The methylation levels of the two CpG islands were detected by BSP. The results showed that the upstream sequence from -258 bp to +31 bp (p2) had no obvious variation in the methylation level between the MG and healthy groups. The ATG upstream sequence from -666 bp to -237 bp (p1) was clearly downregulated in MG patients compared with in healthy individuals. The methylation level of the p1 promotor was 12.25% in the PBMCs from healthy controls and downregulated to 5.16% in the MG patients (*p* < 0.05) (Figure 2(c)). Moreover, the pyrosequencing results of samples from 64 MG patients and 64 healthy volunteers showed a downward trend of the methylation level of each CpG site in p1 (Figures 2(b)–2(d)).

**Figure 3:**
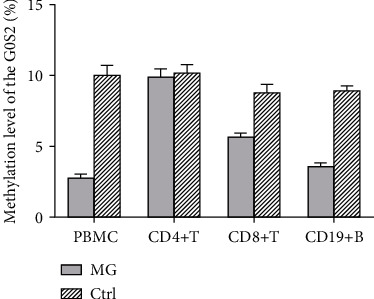
The methylation level of the G0S2 in different type of cells. The *y*-coordinate represents the rate of methylation result; MG means the PMBC sample from myasthenia gravis patients; control means PMBC sample from healthy volunteers. CD4+ T cell and CD19+ cells were different types of cells derived from MG PMBC sample.

The total PBMCs from the control group had a 10% methylation level on the *G0S2* promoter, while the PBMCs from the MG group had a 3% methylation level on the *G0S2* promoter. It is worth mentioning that the methylation level of the *G0S2* promoter in different cell types showed a different pattern (Figure 3). The methylation level of *G0S2* in the CD4+ T cells, CD8+ T cells, and CD19+ B cells between the MG patients and healthy volunteers were analyzed, and positive results were obtained. The CD 19+ B cells from the control group had an 8.8% methylation level on the *G0S2* promoter, while the CD19+ B cells from the MG group had a 3.44% *G0S2* methylation level. The CD4+ T cells from the MG patients had a 9.76% DNA methylation level, which was equivalent to the methylation level of the CD4+ T cells from the healthy controls (9.88%). The same result was obtained for the CD8+ T cells (8.62% for the MG group vs. 9.12% for the healthy volunteers). These results indicate that the changes in the methylation level of the *G0S2* DNA promoter from PBMCs were from B cells and CD8+ T cells while the CD4+ T cell showed little change.

**Figure 4:**
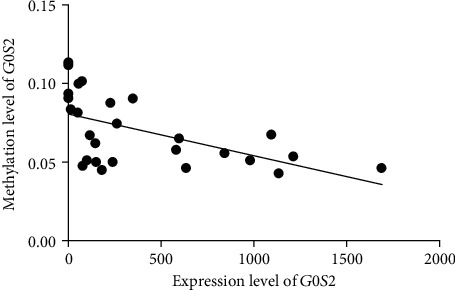
The correlation analysis between the expression level and methylation level of the G0S2. 95% confidence interval is -0.8295 to -0.3475 The *y*-coordinate represents rate of methylation result; each dot represents an individual expression result ofG0S2. The oblique line is the fitting curve of methylation level of G0S2.

It is known that the methylation level of the DNA promoter region is inversely correlated with the expression level of the target gene. These results suggested that the lower methylation level of p1 may increase the expression of *G0S2* in MG patients. We also detected the expression level of *G0S2* in these samples, and a significant negative correlation was found between the expression level of the *G0S2* gene and the methylation level of the *G0S2* promoter (Figure 4 and Table 2). The expression of *G0S2* in CD19+ B (*p* < 0.05) and CD8+ T (*p* < 0.05) cells from MG was upregulated comparing with that from healthy control, but the expression the *G0S2* in CD4+ T cell showed no significant change between 2 groups (*p* > 0.05). This result explains why the methylation level of the promoter decreased while the expression level increased.

### 3.3. NFAT5 Inhibits the Expression Level of *G0S2*

**Figure 5:**
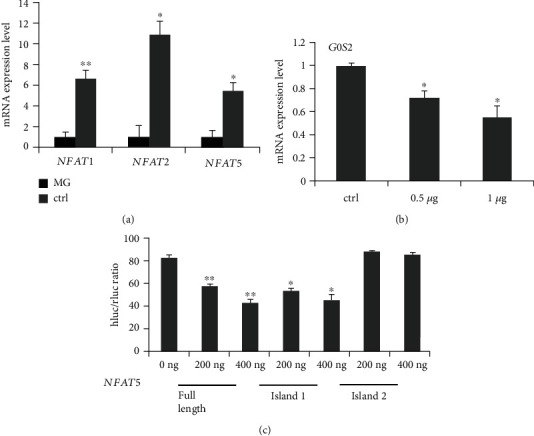
NFAT5 inhibit G0S2 expression through island 1. (a) The relative mRNA expression level of NFAT family in healthy human and MG patients. The *y*-coordinate represents the relative expression level, MG means the myasthenia gravis patients, and ctrl means healthy volunteers. (b) Upregulation NFAT5 inhibits G0S2 expression. The *y*-coordinate represents the relative expression level of G0S2; the *x*-coordinate represents the different concentrations P-EGF-N1-NFAT5 plasmid which were transfected into T cells. (c) NFAT5 binds island 1 of G0S2 promoter region detected by dual-Luciferase reporter. Full length represents the plasmid containing islands 1 and 2; islands 1 and 2 represent the plasmid containing the corresponding CpG island sequence from G0S2 promoter.

**Figure 6:**
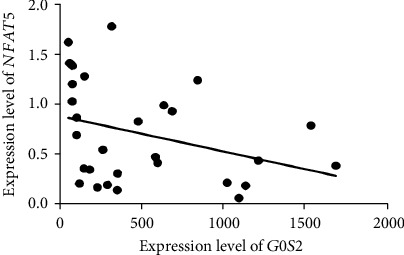
The correlation analysis between the expression level of G0S2 and NFAT5. The *y*-coordinate represents expression level; each dot represents an individual expression result of G0S2. The oblique line is the fitting curve of expression level of NFAT5.

The transcription factor gene family NFAT consists of five different members, including *NFATc1*, *NFATc2*, *NFATc3*, *NFATc4*, and *NFAT5*. To verify the relationship between the NFAT family members and *G0S2*, the expression levels of the five NFAT members in MG patients were determined by qRT-PCR. We found that *NFATc1*, *NFATc2*, and *NFAT5* were downregulated in the PBMCs of MG patients (Figure 5(a)). In particular, there was a negative correlation between the expression level of *NFAT5* and the expression level of *G0S2* (Figure 6). To verify the relationship between *NFAT5* and *G0S2*, the recombinant plasmid pEGFP-N1-NFAT5 was constructed and transfected into PBMCs from MG patients. The expression level of *G0S2* after *NFAT5* overexpression was decreased by 2-fold. Moreover, the fold change depended on the concentration of the pEGFP-N1-NFAT5 recombinant plasmid (inserted next to *NFAT5*) (Figure 5(b)). The expression level of *G0S2* decreased to 0.8-fold of that of the control group when the concentration of the recombinant plasmid (inserted by *NFAT5*) was 0.5 *μ*g and decreased to a half the control group level when the concentration was 1.0 *μ*g. However, according to the BSP results, the methylation status of *G0S2* in transfected PBMCs from MG patients was different from that in nontransfected PBMCs from MG patients. This result suggests that NFAT5 could downregulate the expression level of *G0S2* but not by methylating the promoter of the target gene.

### 3.4. NFAT5 Regulates *G0S2* by Binding to p1 in the Promoter

Our results suggest that *NFAT5* could downregulate the expression level of *G0S2*. To explain the regulatory mechanism, the recombinant plasmids pGL3-Basic-p1 (inserted by p1), pGL3-Basic-p2 (inserted by p2), pGL3-Basic-p12 (inserted by continuous p1 and p2 sequences), and pEGFP-N1-*NFAT5* (inserted by *NFAT5*) were constructed. A dual-luciferase reporter system was employed to reveal the interaction between the *NFAT5* gene and the *G0S2* promoter. With increasing concentrations of pEGFP-N1-NFAT5 transfected into Jurkat T cells (pGL3-Basic-p1-, pGL3-Basic-p2-, and pGL3-Basic-p12-containing cell lines), the fluorescence of pGL3-Basic-p1- and pGL3-Basic-p12-containing cell lines progressively decreased (Figure 5(c)). The pGL3-Basic-p2-containing cell lines showed no significant changes. These results suggested that NFAT5 could regulate the expression level of *G0S2* by binding to p1 in the promoter but showed no binding to p2 in the promoter.

### 3.5. Tacrolimus Therapy Reduces the Expression Level of *G0S2* in MG Patients

**Figure 7:**
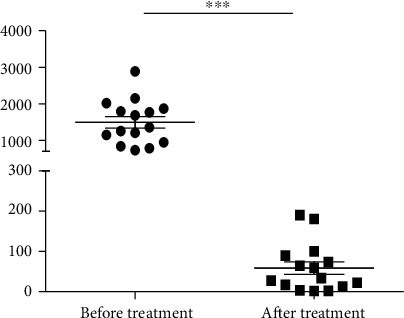
The expression of G0S2 in the PMBC from MG patients before and after treatment with tacrolimus therapy. ^∗∗∗^*p* < 0.01. The *y*-coordinate represents expression level; each dot or blocks represents an individual expression result of G0S2.

Tacrolimus, also named FK-506 or fujimycin, is an immunosuppressive drug that is used to inhibit the immune system for specific reasons, such as an allogeneic organ transplant [8]. It has immunosuppressive properties similar to those of cyclosporin A but is much more potent. To explore the therapeutic effect and mechanism of tacrolimus on MG patients, we measured the expression levels of the *G0S2* transcripts in PBMCs from MG patients before and after three months of tacrolimus therapy using qRT-PCR. The expression level of *G0S2* in MG patients after tacrolimus therapy for three months was generally downregulated compared with the expression level before treatment (Figure 7), demonstrating that tacrolimus therapy could reduce the expression of *G0S2* in MG patients. In addition, the expression level of *NFAT5* was upregulated after treatment with tacrolimus. The expression levels of *NFAT5* in the drug (tacrolimus therapy)-sensitive and nonsensitive groups showed different characteristics; the expression level of *NFAT5* in the drug-sensitive group was upregulated by 2-fold, while that in the nonsensitive groups showed no change.

### 3.6. Both Methylation and Overexpression of *NFAT5* Could Reduce the Expression of *G0S2*

**Figure 8:**
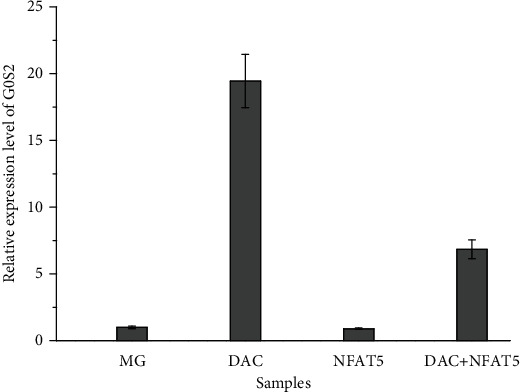
The expression level of G0S2 in the PMBC with different treatment. MG means the PMBC sample from myasthenia gravis patients; DAC means PMBC sample cocultured with DAC(5-aza-dC) to perform DNA demethylation. NFAT5 means PMBC sample which was transfected with P-EGF-N1-NFAT5 plasmid; DAC+NFAT5 means PMBC sample which were transfected with P-EGF-N1-NFAT5 plasmid after coculturing with DAC(5-aza-dC).

After treatment with DAC, the expression level of the *G0S2* gene was upregulated by 20-fold (Figure 8), which means that the expression of *G0S2* in MG patients was controlled by the methylation level of *G0S2*. After demethylation of the *G0S2* promoter, the expression level of *G0S2* was upregulated. Moreover, NFAT5 overexpression downregulated the expression level of demethylated *G0S2* genes.

## 4. Discussion

MG is an old disease caused by immune system disorders that can lead to muscle weakness in patients [8]. At present, the major therapies for MG are cholinesterase inhibitors, immunosuppressants, and glucocorticoid. The change in the expression pattern of *G0S2* and *NFAT5* in the PBMCs of MG patients compared with that in healthy individuals suggests an immune cell disorder. A disruption in the cell cycle of immune cells could be one reason for the immune disorder of MG patients.

The *G0S2* gene was originally discovered in PBMCs and was associated with the cell cycle. *G0S2* expression is required to accelerate the cells into the G1 phase from the G0 phase [21]. Recent studies suggest that G0S2 is a multifunctional protein involved in subcellular localization, gene expression profiling, proliferation, differentiation, and lipid metabolism [22, 23]. In a previous study, the expression level of G0S2 showed an obvious upward trend in the thymus of MG patients [9]. Here, the same result that the expression level of *G0S2* increased in the thymus tissues and PBMCs of MG patients was detected by qRT-PCR. The high expression level of *G0S2* (2000-fold change) in the PBMCs of MG patients and the cell cycle-associated function may suggest that G0S2 could be an important factor in MG.

As for the different cell types, the methylation levels of *G0S2* in CD4+ T cells, CD8+ T cells, and CD 19+ B cell between the MG patients and healthy volunteers were analyzed. The difference between MG patients and healthy controls was most significant in B cells and least significant in CD4+ T cells. MG is directly mediated by autoantibodies produced by B cells, and the presence of pathogenic autoantibodies highlights the importance of B cells in MG pathophysiology, the role of upstream T cells, and their regulation with B cells. The majority of studies on the pharmacodynamic effects of tacrolimus and the NFAT family focus on T cells. This study suggests that NFAT5 and G0S2 may play different roles in different cell subsets.

Different subsets of immune cells that interactively work together are necessary for immune response [24]. MG is an AChR type for B cell-mediated autoimmune disorders. The production of autoantibodies clearly implicates a direct role for B cells in the disease pathogenesis. Dysregulation in immune cell types extending beyond B lineages has been documented, indicating that a combination of factors contributes to disease manifestation. Moreover, the B cell response in MG most certainly requires T cell help. Proinflammatory antigen-specific T cells are involved in AChR MG, and the pathogenic anti-AChR antibodies are high-affinity IgGs, whose synthesis requires interaction of activated CD4+ T cells with B cells that synthesize low-affinity anti-AChR antibodies [25]. Accordingly, CD4 T cells are the drivers in the immunopathogenesis of MG disease, and they play a multifaceted role in immunity, from promoting inflammation to inducing immune tolerance and supporting B cell function. CD8+ T cells are less important to disease pathophysiology in AChR MG [26], although CD8+ T cells from MuSK MG patients had higher frequencies of polyfunctional responses than the controls and CD4+ T cells had higher IL-2, TNF-alpha, and IL-17. In experimental autoimmune encephalomyelitis, CD8+ cells were shown to be both effectors and regulators. CD8+ cells have been implicated as suppressor or regulator T cells in other autoimmune diseases [27]. Recent studies suggest that the functions of T cells are more complicated [28]. CD8+ cells were also found to help B cells in antibody production through the expression of the CD40 ligand [29, 30]. The importance of CD4+ T cells in the pathogenesis of MG is consistent with previous data on experimental autoimmune myasthenia gravis (EAMG) and human MG [31]. However, the role of CD8+ T cells in MG is unclear. Another report, however, showed that both CD4+ and CD8+ cells are essential for the development of EAMG. As the primary site of T cell development, the abnormal immune response of the thymus is closely related to the occurrence of MG [32, 33]. Recent clinical studies have shown that thymus abnormalities might have an impact on the expression of the proapoptotic factor Fas in CD4-CD8+ T cells. Indeed, an imbalance between pathogenic Th17 and Treg cells characterizes the peripheral blood of AChR MG patients and likely contributes to a loss of tolerance. Accordingly, a research group demonstrated that the proportion of Th17 cells and IL-17-producing CD8+ T cells was increased in the prethymectomy peripheral blood of MG patients compared with the controls [34].

Immunosuppressors are the main treatment for MG, especially for those who suffered severe adverse effects of glucocorticoid. Previous studies provided evidence that CYA is effective as a monotherapy or in combination with glucocorticoid in MG [35]. However, cyclosporine A also showed serious side effects. To date, cyclosporine A has been increasingly replaced by a new immunosuppressant, tacrolimus. Tacrolimus showed better efficacy with fewer side effects than cyclosporin A, which downregulated the expression level of G0S2 in cultured blood mononuclear cells by inhibiting the activities of the calcium-dependent phosphatase calcineurin and the NFAT transcription factor family [8, 36, 37] and by suppressing the immune system. A previous study suggested that the *G0S2* gene has potential sites in the 5′ arm that could bind to the NFAT gene [14]. For this reason, NFAT could regulate the expression level of *G0S2* by binding to the promoter region. In this study, all members of the NFAT gene family were detected, and only the *NFAT5* gene showed a negative correlation with *G0S2*. Furthermore, dual-luciferase reporter experiments showed that *NFAT5* could downregulate the expression of *G0S2* by binding to p1 in the promoter region of *G0S2*.

Generally, affecting the promoter region of one gene could result in DNA methylation and downregulate the gene expression level. For the first time, we found that *G0S2* methylation was significantly lower in the PBMCs of MG patients than in those of healthy controls, which indicated that the decreased *G0S2* methylation may be associated with upregulated *G0S2* expression at the mRNA level. Moreover, our work revealed that *NFAT5* could combine with p1 in the *G0S2* promoter to determine *G0S2* expression in the thymus tissues and PBMCs of MG patients. Owing to the abnormal activation of T cells in MG patients, NFAT5 is involved in T cell activation. Our results are consistent with the findings observed in studies on other autoimmune diseases [38]. NFAT5 binding and methylation at p1 in the *G0S2* promoter could control the expression of the *G0S2* gene to regulate the lymphocyte balance. Unfortunately, NFAT5 showed no effect on *G0S2* methylation. NFAT5 may affect the transcription initiation or transcriptional regulation of G0S2 to downregulate its expression. In summary, we speculate that the expression of G0S2 may affect the lymphocyte cell cycle and that G0S2 expression is required to induce cells to transition from the G0 phase to the G1 phase. The interaction of T cell activation factor (NFAT) with G0S2 may affect the number and activation of T cells, thus further affecting its influence on MG.

Like cyclosporine, tacrolimus (FK506) could inhibit the activity of calcineurin with less nephrotoxicity [39, 40]. Several reports have suggested its potential benefit in MG. Tacrolimus is used for the treatment of MG patients who are intolerant to mycophenolate mofetil, azathioprine, and ciclosporin [41]. The expression level of *G0S2* was downregulated after 3 months of tacrolimus therapy. Furthermore, tacrolimus therapy could have a positive effect, generally reducing the expression level of *G0S2* in MG patients. In addition, *G0S2* is recognized as a potential tumor suppressor gene [9], which may explain why the side effects of tacrolimus lead to tumorigenesis. The expression of the *G0S2* gene may play an important role in maintaining the T lymphocyte balance. Therefore, the regulation of *G0S2* methylation can provide more evidence to explain the molecular mechanism of MG and offer new insights into the development of epigenetic-based therapeutic strategies for MG. Further studies are needed to develop new long-term immunosuppressive therapy strategies for MG.

## Figures and Tables

**Table 1:** Primers used in the study.

Primer name	Primer sequence (5′-3′)	Utilization
G0S2-F	GAGAGGAGGAGAACGCTGAG	qRT-PCR
G0S2-R	CTTCTGGGCCATCATCTCCT	qRT-PCR
DNMT3B-F	CAAACCCAACAACACGCAAC	qRT-PCR
DNMT3B-R	ATCTTCCAGGCTGCTCTTGT	qRT-PCR
DNMT1-F	ACCAAGAACGGCATCCTGTA	qRT-PCR
DNMT1-R	GCTGCCTTTGATGTAGTCGG	qRT-PCR
NFAT1-F	AACACCAAAGTCCTGGAGATAC	qRT-PCR
NFAT1-R	AATGTCCGTCTCGCCTTTC	qRT-PCR
NFAT2-F	AATTCTCTGGTGGTTGAGATCC	qRT-PCR
NFAT2-R	TACTGGCTTCGCTTTCTCTTC	qRT-PCR
NFAT5-F	GATTCAGCCCAAGGCATACA	qRT-PCR
NFAT5-R	GCAGCTGACTAGAAGCAGAAA	qRT-PCR
NAPDH-F	GCCAAAAGGGTCATCATCTC	qRT-PCR
NAPDH-R	GTAGAGGCAGGGATGATGTTTC	qRT-PCR
G0S2-bsp-1F	TAATGTTAGGTTGTTTTGGATAAGG	BSP PCR
G0S2-bsp-1R	ACTACAACTCTCCCAATTAAAAACTC	BSP PCR
G0S2-bsp-2F	TTTTAATTGGGAGAGTTGTAGTTGT	BSP PCR
G0S2-bsp-2R	ACCAAAAAAATCAACTCCTAAACC	BSP PCR

**Table 2:** Fold change of G0S2 and methylation level in PBMC from the same MG patients.

	Fold change of G0S2 in PBMC from MG	Methylation level in PBMC from MG		Fold change of G0S2 in PBMC from MG	Methylation level in PBMC from MG
MG1	839.8262	0.05586734	MG14	3.16	0.09028552
MG2	261.11	0.07480559	MG15	1132.38	0.04281364
MG3	226.86	0.0878684	MG16	348.11	0.09049833
MG4	117.06	0.06729902	MG17	238.84	0.05023203
MG5	48.95	0.08162508	MG18	145.13	0.06219874
MG6	72.44	0.1017963	MG19	977.65	0.05075296
MG7	1.5	0.1118319	MG20	595.15	0.06516683
MG8	99.88	0.05115332	MG21	1211.064	0.05382841
MG9	147.86	0.05028404	MG22	1684.133	0.04635253
MG10	74.85	0.04752094	MG23	1091.28	0.06756885
MG11	14.06	0.08366191	MG24	632.5179	0.04639083
MG12	581.65	0.05811148	MG25	181.0193	0.04508229
MG13	56.93	0.1000311	MG26	1.940391	0.1133023
			MG27	2.623941	0.09335699

## Data Availability

All data, models, and code generated or used during the study appear in the submitted article.
